# Initial description of the novel handheld wireless ultrasound device TE Air with Doppler and Color Duplex imaging

**DOI:** 10.3233/CH-238100

**Published:** 2024-02-22

**Authors:** Ernst Michael Jung, Friedrich Jung, Yi Dong, Ulrich Kaiser

**Affiliations:** a Institute for Diagnostic Radiology and Interdisciplinary Ultrasound, University Hospital Regensburg, Regensburg, Germany; b Institute of Biotechnology, Molecular Cell Biology, Brandenburg University of Technology, Senftenberg, Germany; c Xinhua Hospital affiliated to Shanghai Jiaotong University School of Medicine, Shanghai, China; d Medical Clinic and Polyclinic III, University Hospital Regensburg, Regensburg, Germany

**Keywords:** Mindray, handheld ultrasound, TE Air, ultrasound, point-of-care ultrasound

## Abstract

**OBJECTIVE:**

To test and initially describe a new handheld wireless ultrasound technique (TE Air) for clinical use.

**METHODS:**

In this pilot study, the new ultrasound device TE Air from Mindray was used to examine the hepatic and renal vessels of healthy volunteers for first impressions. The probe has a sector transducer with a frequency range of 1.8–4.5 MHz. The B-mode and color-coded doppler sonography (CCDS) scanning methods were used. A high-end device from the same company (Resona 9, Mindray) was used as a reference. The results were evaluated using an image rating scale ranging from 0 to 5, with 0 indicating not assessable and 5 indicating without limitations.

**RESULTS:**

Altogether, 61 participants (*n* = 34 female [55.7%], *n* = 27 male [44.3%]), age range 18–83 years, mean age 37.9±16.5 years) could be adequately studied using TE AIR and the high-end device. With one exception, the image quality score for TE Air never fell below 3 and had a mean/median scored of 4.97/5.00 for the B-mode, 4.92/5.00 for the color flow (CF) mode, and 4.89/5.00 for the pulse wave (PW) mode of the hepatic vein, 4.90/5.00 for the portal vein, 4.11/4.00 for the hepatic artery, and 4.57/5.00 for the renal segmental artery. A significant difference in the assessment of flow measurement of the hepatic artery and renal segmental arteries was found between TE AIR and the high-end device.

**CONCLUSIONS:**

TE Air represents a new dimension in point-of-care ultrasound via wireless handheld devices. Especially, its flow measurement ability offers a relevant advantage over other available handheld models. TE Air provides a formally sufficient image quality in terms of diagnostic significance.

## 1 Background

Ultrasound diagnostics represents an important cornerstone of imaging diagnostics in many medical fields [1, 2]. Using the latest high-end ultrasound techniques, highly effective means for abdominal diagnostics are available [3–7]. Apart from the very high equipment costs, the size and bulkiness of these devices (e.g., limited freedom of movement of the monitor screen) pose a challenge that should not be underestimated [8, 9]. The hygienic reprocessing of the devices after use also requires increased commitment in terms of time for correct implementation. Oftentimes, battery operation is not yet possible or these devices can only be used in very limited time.

With the development of new technology of wireless handheld ultrasonography, several ultrasound devices exist which could address the abovementioned disadvantages. Especially, the “pocket-size” and the cable connection replacement between the ultrasound probe and monitor by a wireless connection make these devices very interesting and user-friendly [9–11]. During pandemics, such as the recent coronavirus disease pandemic, these easy-and-quick-to-disinfect ultrasound models could bring a decisive advantage. First indications of sufficient image quality, of the integrated CF mode, and of the operational capability have already been shown in several studies, including our previous investigations [3, 8, 9, 12].

However, these devices lack the ability to assess hemodynamics with flow velocity measurements in pulse wave mode [12]. With the new technology of the TE Air, a wireless handheld ultrasound device (HHUD) is now available for the first time, which has this technician extension. However, clinical evaluations of liver and kidney perfusion using the TA Air technique have not been performed yet.

The present pilot study aimed to investigate in a first description the outcome of the novel TE Air device with focus on testing the PW mode in relation to liver and kidney perfusion.

## 2 Methods

### 2.1 Study design

In May 2023, diagnostic examinations were performed in a university ultrasound center in Germany using the new point-of-care ultrasound technology TE Air from the company Mindray. Survey participation was voluntary and anonymous. A high-end device (Resona 9, Mindray, China) was available as a reference. Both high-end and mobile devices were developed by the same manufacturer; thus, it can be assumed that the two systems are comparable.

The investigations were carried out with the approval of the local ethics committee without any ethical objections.

The ultrasound examinations were performed by an experienced examiner (more than 3000 examinations/year, more than 30 years of experience in sonography, DEGUM III certification). The examination procedures were conducted in the same manner for both ultrasound devices used. Image quality was evaluated off-line by two experienced examiners, each with at least 5 years of ultrasound examination experience.

The collected data were then transferred to the program IBM SPSS Statistics version 29.0.0.0 (241) for further analysis.

The evaluation was performed both descriptively and by means of special hypothesis testing.

### 2.2 Participants

Participants with healthy liver and kidney were enrolled to avoid the influence of potential diseases on the evaluation of image quality and flow measurements of the liver and kidney vessels. If a pathology affecting the measurements was detected, this participant was excluded. Moreover, participants with obesity were excluded to minimize influence on image quality. Participants were selected regardless of age or sex.

### 2.3 High-end device

The Resona 9 model (Mindray, China) with an SC6-1U multi-frequency transducer (1.2–6.0 MHZ) was used. When anatomically possible, the entire affected region was examined. Contrast ultrasound was not used. The images were stored anonymously in our Picture Archiving and Communication System (PACS).

Color-coded doppler sonography (CCDS) with digital storage of pulse wave Doppler, Color Duplex, and high-resolution B-mode were used in addition to the B-mode.

### 2.4 Point-of-care ultrasound (POCUS)

**Fig. 1:**
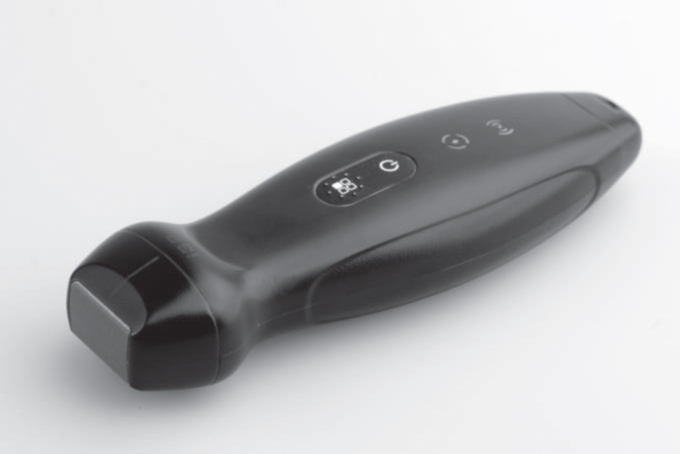
Illustration of the TE Air device.

**Fig. 2:**
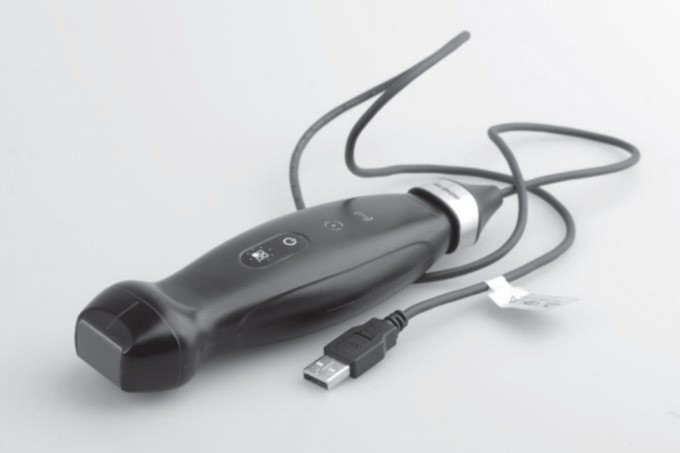
Illustration of the coupling mechanism between the TE Air device and charging cable.

The sector probe (1.8–4.5 MHz) of the HHUD TE Air (Fig. 1) (Mindray, China) was used. This new type of wireless ultrasound device, in addition to the classic B and CF modes, has additional settings, such as a PW mode or power mode. The wireless connection between the ultrasound probe and Apple device used (iPhone 14, iOS version 16.2, Apple, USA) was obtained via a protected WLAN connection. It is a mobile iOS device with touchscreen on which the corresponding TE Air application program has been installed and applied. The HHUD’s battery could be charged on site at any time using a suitable charging cable (Mindray, China) by rapid charging (duration of use on average: approximately 5 min per participant), minimizing downtimes and delays between examinations. The connection mechanism was based on a magnetic coupling system (Fig. 2). Additionally, the TE Air has a waterproof and dustproof design according to IP68 as well as sufficient possibilities for disinfection due to its nature.

The images obtained were also stored anonymously and password-protected — in compliance with the applicable data protection regulations — on the mobile device used.

### 2.5 Image quality

The image quality was evaluated using a scoring system ranging from “0” to “5”, with 0 indicating no evaluation possible (e.g., due to very heavy artifacts); 1, no diagnostic evaluation possible; 2, high degree of diagnostic assessment limitations (no reliable assessment); 3, proportionate limitations in diagnostic evaluation, but sufficient for reliable assessment (e.g., due to mild edema); 4, only mild limitations of the diagnostic evaluation; and 5, excellent image quality without limitations.

### 2.6 Examination procedure

Participants were examined in the supine position after resting for > 10 minutes. First, the right intercostal section was used to visualize the hepatic hilum area with focus on the portal vein and hepatic artery in B-mode. Subsequently, the CCDS mode was added. After visualization of the portal and hepatic veins and hepatic artery using this mode, flow measurement was performed in the following sequence: portal vein ->hepatic vein ->hepatic artery. Second, the examination of the right kidney was also initially set in B-mode in the right flank section. Subsequently, the CCDS mode was added, and the flow measurement of the renal segmental artery that was best visualized was performed.

### 2.7 Statistical methods and analysis

IBS SPSS Statistics version 29.0.0.0 (241) was used for the statistical analyses. The Gaussian normal distribution of the examined data was tested by Shapiro–Wilk test and, due to the small number of cases, graphically by means of histogram and Q–Q plot. If a normal distribution could be assumed, the hypothesis test of the paired samples was performed by paired *T*-test; in case of non-fulfillment, the test was performed by using the Wilcoxon test. The significance level was set at 1% (*α*=  0.01).

## 3 Results

Altogether, 61 participants (*n* = 34 female [55.7%], *n* = 27 male [44.3%]; age range 18–83 years, mean age 37.92±16.53 years) were enrolled (Table 1). Adequate examination conditions were possible for each participant using high-end and TE Air Device. In all cases, the hepatic (portal vein, hepatic vein, and hepatic artery) and renal vessels could be visualized. The average examination time was 5 minutes per participant.

**Table 1:** Epidemiological data of the participants

Variable	Number
Total number	61 (100%)
Sex
Female	34 (55.7%)
Male	27 (44,3%)
Age
>18 Years	61 (100%)
Mean age	37,92 (±16,53)
Age range	18–83 (Years)

With the high-end device, the image quality and measurement results of the hepatic and renal vessels were consistently good to very good, except in one case. Relevant restrictions during the examination were not observed.

With the TE Air device, the image quality score never fell below 3, except in one case (Tables 2, 3, and 4). Nineteen participants obtained a score of 3, with isolated cases involving more than one structure. The following structures were affected: hepatic artery (*n* = 18), hepatic vein (*n* = 1), and renal segmental artery (*n* = 2). In cases with a complicated anatomical course, especially of the hepatic artery, the measurement results were affected.

**Table 2:** Imaging of the hepatic vessels using the TE Air device

TE Air device	Description
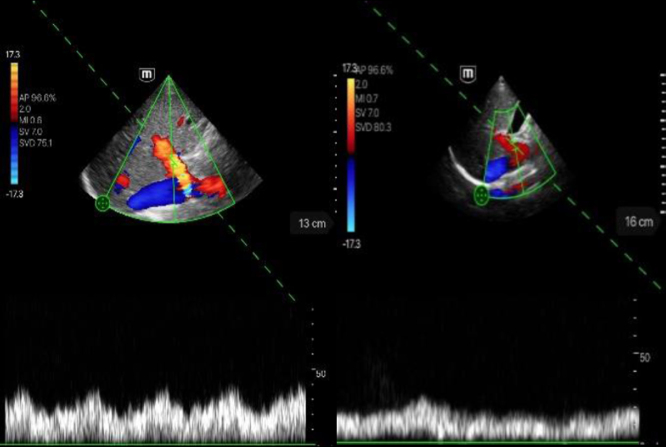	Visualization of the portal vein in the intercostal section. Left image: score 5; hepatopetal flow, no stenoses or thromboses. Right image: score 4; hepatopetal flow, no stenoses or thromboses.
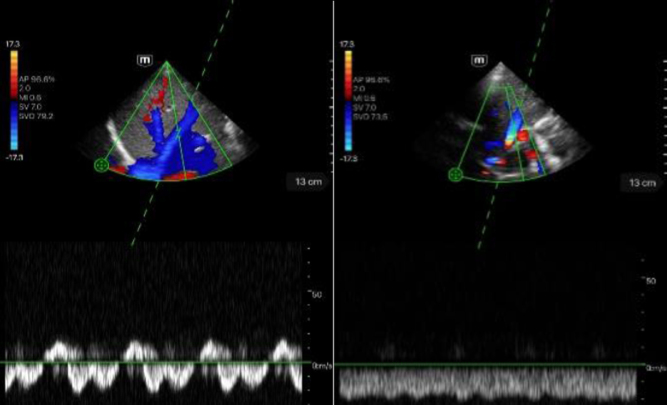	Visualization of the hepatic vein in intercostal section. Left image: score 5; triphasic flow profile. Right image: score 4; still biphasic flow profile.
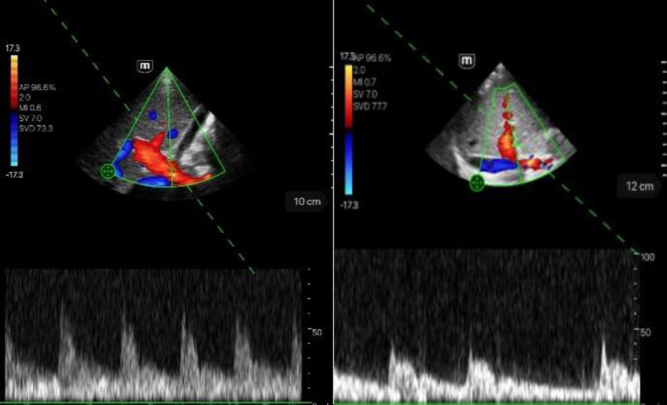	Visualization of the hepatic artery in intercostal section. Left image: score 5; biphasic without aliasing, flow systolic. Right image: score 4; biphasic without aliasing, flow systolic.

**Table 3:** Imaging of the renal artery vessels using the TE Air device

TE Air device	Description
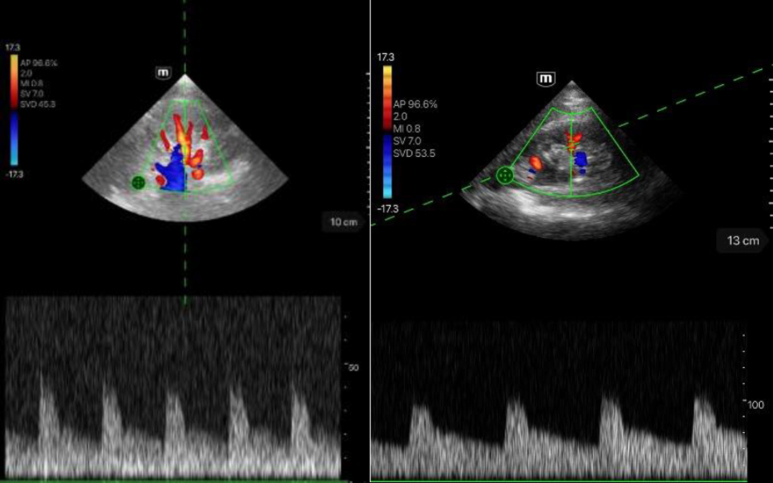	In the images, a good to very good image quality of the flow derivation of the renal arterial vessels could be achieved. The pulsatile flow course can be seen clearly and very well delineated. Left image: score 5. Right image: score 4.

**Table 4:** Imaging of the hepatic vessels and kidneys in B- and CF-modes using the TE Air device

Portal vein	Description
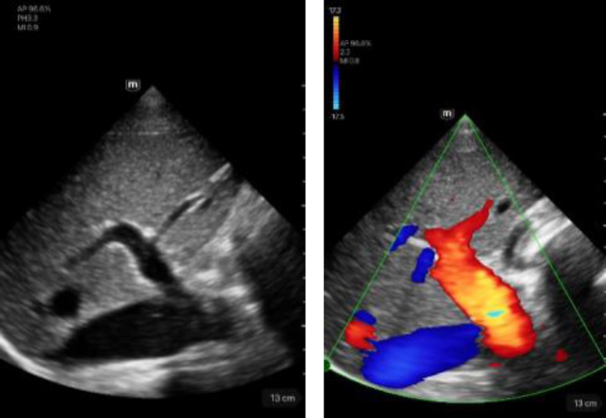	Left image: B-mode visualization of the portal vein in intercostal section. Score 5. Right image: showing the portal vein in CF mode in intercostal section. Score 5.
Hepatic vein	Description
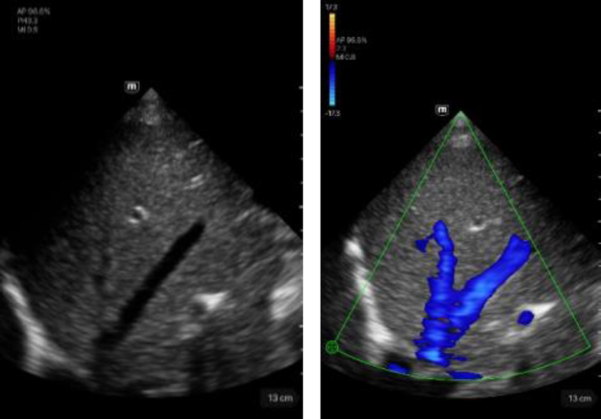	Left image: B-mode view of the hepatic vein in intercostal section. Score 5. Right image: showing the hepatic vein in CF mode in intercostal section. Score 5.
Hepatic artery	Description
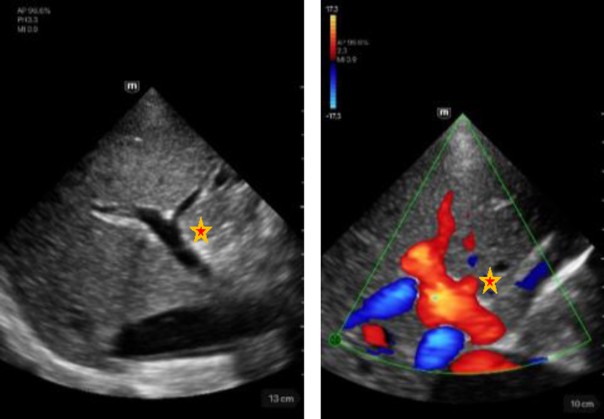	Left image: B-mode view of the portal vein with adjacent hepatic artery (marked by a star) in intercostal section. Score 5. Right image: Illustration of the portal vein with adjacent hepatic artery (marked by a star) in CF mode in intercostal section. Score 5.
Kidney	Description
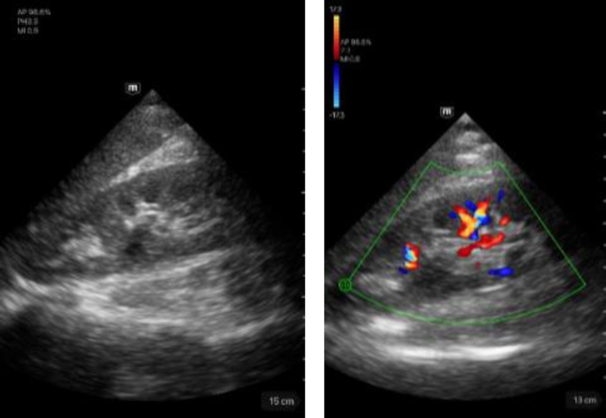	Left image: B-mode imaging of the right kidney. Score 5. Right image: imaging of the right kidney in CF mode. Score 5.

Table 5 shows the medians and mean values of the measurement results of the ultrasound devices used.

**Table 5:** Quality of the visual image of the examined structure by using a high-end device and the TE Air device. Shown is the mean value including the standard deviation and calculated significances in the hypothesis test

Examined structure in PW mode	High-End device	TE Air device	*p*-Value
Portal vein	5.00 (±0.00)	4.90 (±0.30)	0,014
Hepatic artery	4.92 (±0.33)	4.11 (±0.88)	<0,001
Hepatic vein	5.00 (±0.00)	4.89 (±0.37)	0,02
Renal segmental artery	4.87 (±0.34)	4.57 (±0.56)	<0,001

In all cases, we observed, in the three intrahepatic vein areas, a bi- to triphasic flow profile with a Vmax of up to 30 cm/s (mean of 22±5 cm/s), an A. hepatica flow of up to 90 cm/s (mean of 78±7 cm/s), a diameter of the V. portae of ≤1 cm, and a flow of up to 42 cm/s (mean of 26±5.5 cm/s).

Significant differences in the measurements of the hepatic artery and renal segmental arteries were observed between the two devices (hepatic artery *p*-Value = <0,001; renal segmental artery *p*-Value = <0,001). The B-scan, CF mode, and portal and hepatic vein measurements showed no significant results (Table 5).

## 4 Discussion

The field of mobile ultrasound continues to develop. This includes the availability of new ultrasound models, such as the wireless HHUS devices. In this first description we aimed to investigate the outcome of the novel TE Air device with focus on testing the PW mode in relation to liver and kidney perfusion.

The available initial results appear promising for the new TE Air mobile ultrasound system. Its penetration depth and image quality in B-mode prove to be consistently good to very good as compared to those of the high-end system. With color doppler (CCDS), a hemodynamic assessment with the TE Air can always be performed with at least a good image quality. This is especially true for the hepatic and portal veins where no significant difference was seen in the cohort studied, even in cases with deeply positioned vessels of up to approximately 15 cm. Kidney perfusion was also feasible with at least good quality to a depth of approximately 15 cm. This can best be explained by the existing frequency range of the ultrasound probe (1.8–4.5 MHz). In the evaluation of the hepatic and renal segment arteries, the high-end device is significantly superior, but the image quality of the TE Air is also at least good.

The advantage of the high-end system is the possibility of using the high-resolution convex probe with matrix technology (C1-6), unlike the sector probe of the TE Air. Additionally, the high-end system at CCDS also has the additional technology of Glazing flow and high flow with markedly improved flow detection, which has also been shown in our own investigations [5]. The examiner’s experience plays a decisive role not only in B-mode, but also especially in CCDS. High-end systems offer a detailed adjustment of the flow parameters via scale, pulse repetition frequency (PRF), wall filter, color gain, and dynamic range. Furthermore, auto-optimization is also present [13]. Therefore, the high-end device has an edge over the TE Air. This also confirmed the poorer image quality of the TE Air device, as compared to that of the high-end device, in cases with complicated anatomical anatomy and course of the hepatic artery, which were much more difficult to image with the handheld system.

In the comparison of different ultrasound systems, if possible, comparable ultrasound sections, for example, in the liver from the transcostal or intercostal section or in the kidney from obliquely angulated or in the flank section, should be included. The easy-to-handle sector probe can offer advantages in the detection process. Particularly in the intercostal section, the sector probe provided a good localization of the hepatic vessels and reduced the image impairment due to the rib shadows. Nevertheless, the relevant advantages of the high-end system include autofocus and auto-optimization of the B-mode, CCDS optimization, and depth adjustment.

With the ability to visually assess the hepatic vasculature with the TE Air system with at least good image quality, mobile imaging now allows the assessment of a variety of issues related to vascular changes in the liver. The present pilot study initially captures predominantly still normative evaluations of the portal and hepatic veins and hepatic artery. For renal perfusion, the focus was on recording the segmental perfusion. This is a decisive factor for the detection of reduced blood flow or the clarification of a possible renal hypertension.

The present study represents a monocentric pilot study; thus, it has some limitations. In addition to the small number of participants, only healthy participants were tested. No assessment of pathological structures was performed. Particularly, the pathology assessment power of this new generation of handheld devices is an interesting aspect, as previous generations of devices were not comparable to high-end ultrasound devices [14]. No comparison was made with other handheld models. Likewise, the applicability in clinical routine was not specifically investigated. This should be investigated in further studies. Given that only the sector probe is available so far, some structures, especially the cervical vessels or superficial structures, can only be assessed to a limited extent. Other model variants with further probe types, including linear or convex probes, would be useful.

In conclusion, the TE Air device’s small size, easiness of use, and especially the possibility of variable image transmission to an external monitor represents major advantages for various areas, including teaching, which is in line with the results of previous studies [8, 10]. The device’s simple and fast disinfection is also a relevant advantage — especially during pandemics such as the recently survived COVID-19 pandemic — and enables safer and adequate patient care. This complements existing literature that demonstrated the advantages of HHUDs [15, 16]. However, as promising as these initial results appear, further investigation of the abdominal vascular changes is still needed, including multicenter verification of these changes.

## Conflicts of interests

The authors have no potential conflicts of interest to declare.
